# 0733. Impact of endotoxin challenge on disseminated intravascular coagulation in obese minipigs

**DOI:** 10.1186/2197-425X-2-S1-P55

**Published:** 2014-09-26

**Authors:** T Duburcq, A Tournoys, F Pattou, T Hubert, V Gmyr, L Quintane, R Favory, J Mangalaboyi, M Jourdain

**Affiliations:** INSERM, U859 Lille, France; European Genomic Institute for Diabetes (EGID), Lille, France; Pole de Réanimation CHRU, Lille, France; Centre de Biologie Pathologie CHRU, Lille, France

## Introduction

An early activation of coagulation and fibrinolysis occurs during sepsis and results in a marked increase of thrombin and fibrin formation, leading to the syndrome of disseminated intravascular coagulation (DIC). DIC is a strong predictor of death and multiple organ failure in patients with septic shock [1]. Obesity has been demonstrated to be a hypercoagulable [2] and hypofibrinolytic [3] state but its impact on coagulation and fibrinolysis during sepsis has never been studied.

## Objectives

In this study, we aimed to determine if obesity impairs DIC in an acute endotoxic shock using minipigs.

## Methods

This was a prospective, comparative and experimental study, approved by the Animal Ethics Committee. Pigs were chosen as a clinically relevant species, resembling to humans in coagulation reactions. Four groups of five “Yucatan” minipigs were studied: lean and obese control groups, lean LPS group receiving Escherichia Coli endotoxin (LPS) and obese LPS group receiving the same endotoxin dose. We measured standard coagulation parameters [prothrombin time (PT), platelet count and fibrinogen levels], thrombin-antithrombin complex (TAT), tissue plasminogen activator (t-PA) and plasminogen activator inhibitor-1 (PAI-1). All measurements were performed at baseline and at 30, 60, 90, 150 and 300 minutes. Results were given as median with 25-75 interquartile ranges.

## Results

At baseline, platelet count (477 [428-532] vs. 381 [307-442] G/l; p=0.005) and fibrinogen levels (4.6 [3.8-5.2] vs. 2 [1.8-2.9] g/l; p< 0.001) were significantly higher whereas prothrombin time (80 [76-92] vs. 96 [89-100] %; p=0.01) was significantly lower in obese pigs compared to lean pigs. Control groups remained stable during the study-period. In LPS groups, administration of endotoxin resulted in a typical hypokinetic shock with DIC. The decrease in coagulation parameters (PT, platelet count and fibrinogen levels) and the increase in TAT complex (581 [382-1057] vs. 247 [125-369] µg/ml at 150 min; p=0.03) were significantly more important in obese LPS group compared to lean LPS group. Concerning the fibrinolytic reaction, we found a more important increase of PAI-1 in obese LPS group at 300 min (481 [365-617] ng/ml vs. 355 [209-660] ng/ml; p=0.66) without reaching statistical significance. Nevertheless, the increase of t-PA was significantly lower in obese LPS group compared to lean LPS group at 90 min (10 [8-17] vs. 5 [2-9] ng/ml; p=0.04).

## Conclusions

In our model of endotoxic shock, obese pigs developed a more severe disseminated intravascular coagulation with a more serious procoagulant response.
